# Most women diagnosed with cervical cancer by a visual screening program in Tanzania completed treatment: evidence from a retrospective cohort study

**DOI:** 10.1186/1471-2458-14-910

**Published:** 2014-09-03

**Authors:** Andrew C Gard, Amr S Soliman, Twalib Ngoma, Julius Mwaiselage, Crispin Kahesa, Robert M Chamberlain, Siobán D Harlow

**Affiliations:** University of Michigan School of Public Health, Ann Arbor, MI USA; University of Nebraska Medical Center College of Public Health, Omaha, NE USA; Ocean Road Cancer Institute, Dar es Salaam, Tanzania

**Keywords:** Cervical cancer, Screening, Visual inspection with acetic acid, Referral patterns, Radiation therapy, Tanzania

## Abstract

**Background:**

Visual inspection with acetic acid (VIA) to identify and treat pre-cancerous lesions is effective for cervical cancer prevention. Screening programs also facilitate screening and diagnosis of invasive cancers that must be referred for radiation therapy or chemotherapy. This study compared characteristics of women diagnosed with invasive cervical cancer by a VIA screening program who did and did not follow up for treatment and who did and did not complete treatment at the Ocean Road Cancer Institute (ORCI), Dar es Salaam, Tanzania.

**Methods:**

We conducted a retrospective cohort study of ORCI screening referrals from the period November 2002 to June 2011. Women referred for treatment of invasive disease (n = 980) were identified from an existing database of all women attending the screening clinic during this period (n = 20,131) and matched to a dataset of all cervical cancer patients attending ORCI in this period (n = 8,240). Treatment information was abstracted from patient records of women who followed up. Records of a random sample (n = 333) of unscreened patients were reviewed for disease stage.

**Results:**

Of the 980 women referred women, 829 (84.6%) sought treatment. Most of those women (82.8%) completed their prescribed radiation. Lower disease stage, having a skilled occupation, residence in Dar es Salaam, and younger age were independently associated with loss to follow-up. Higher disease stage, residence in Dar es Salaam, older age, and later year of first treatment appointment were independently associated with incomplete treatment among those who followed up. Significantly more screened women had stage 1 disease (14.0%) than unscreened women (7.8%).

**Conclusions:**

Most women referred from the screening clinic completed treatment for their cancer at ORCI. Some of those lost to follow-up may have sought treatment elsewhere. In most cases, the screening clinic appears to facilitate diagnosis and treatment, rather than screening, for women with invasive cervical cancer.

## Background

East Africa has the highest cervical cancer burden worldwide [1]. In Tanzania, it is the most common cancer in women, with an estimated annual incidence of 50.9 cases per 100,000 and mortality of 37.5 women per 100,000 in 2008 [1].

Although Pap smear is the most commonly used cervical screening modality in the developed world, it requires substantial medical infrastructure, training, and financial resources to implement, and therefore is not feasible for widespread application in most developing countries [2]. A simpler, more cost-effective [2] screening method is visual inspection with acetic acid (VIA), which has been shown to be accurate for detection of precancerous and cancerous cervical lesions [3] and effective for cancer prevention in large, randomized studies conducted in developing countries [4, 5].

The benefits of VIA are primarily from pre-clinical detection of pre-cancerous lesions treatable with cryotherapy, LEEP, or cold-knife conization, preventing these lesions from progressing to invasive cancer [2, 5]. However, VIA screening programs also facilitate detection of asymptomatic invasive cervical cancers and diagnosis of symptomatic invasive cancers, through both VIA itself and the provision of basic gynecological care by competent health workers. These cancers must be referred for surgery, radiation therapy, or chemotherapy. We have identified no study to date that has provided a comprehensive evaluation of the outcomes of women with invasive cancer detected or diagnosed by a VIA screening program.

Several studies have reported the percentage of women diagnosed with invasive cervical cancer at screening [6–10], with at least four studies offering some information about the treatment received by women referred from screening [11–14]. One study compared the stage of invasive cancer diagnosed in screened and unscreened women [5]. In Mali, 73.4% of 497 women referred from screening received some treatment – 35.0% radical hysterectomy, 34.4% cyclophosphamide chemotherapy, and 4.0% radiation (none completed a full course) [11]. In Angola, 80.7% of 57 women received some treatment – 71.9% surgery or radiation and 8.8% “less-appropriate treatment” [13].

In a randomized study in south India, 82.6% of 69 screening-detected invasive cancers were treated [15]. This is the only study to assess predictors of compliance with treatment after VIA, although the analysis combined cervical intraepithelial neoplasia and invasive cancer. The authors found younger age, practicing some form of contraception, and having a high-grade precursor lesion or invasive cancer (vs. a low-grade precursor lesion) were positively and independently associated with treatment compliance [15]. This study also found that significantly more screened than unscreened women were diagnosed with stage 1 disease (19.8% vs. 10.1%, p = 0.015) [5], an important benefit of screening.

The fourth study demonstrated the feasibility and accuracy of visual inspection and clinic-based treatment of pre-cancerous lesions at the Ocean Road Cancer Institute (ORCI) in Dar es Salaam, the largest city in Tanzania [12]. Women were actively recruited from Dar es Salaam for participation, but this was not a population-based effort. Compiling data from November 2002 to August 2007, 1.9% of the 10,378 women included in the analysis had invasive cancer and 96% were treated [12]. Following the completion of that study in August 2007, ORCI has continued to offer screening on a daily basis [12], but women are generally not actively recruited for screening. This screening program presented an opportunity to explore the referral, treatment, and disease stage patterns of women diagnosed with invasive cervical cancer by a VIA screening program across an extended time period.

We conducted a retrospective cohort study building upon the ORCI study just described. We compared characteristics of women referred for treatment who followed up to those lost to follow-up, of women who completed treatment to those who did not, and the cancer stage of screened and unscreened women who sought treatment. We hypothesized age, disease stage, distance from treatment facilities, socioeconomic status, and reproductive factors would influence follow-up from screening to treatment and treatment completion. We also hypothesized women who attended the screening clinic would be more likely to be diagnosed with stage 1 disease than unscreened women.

## Methods

**Figure 1:**
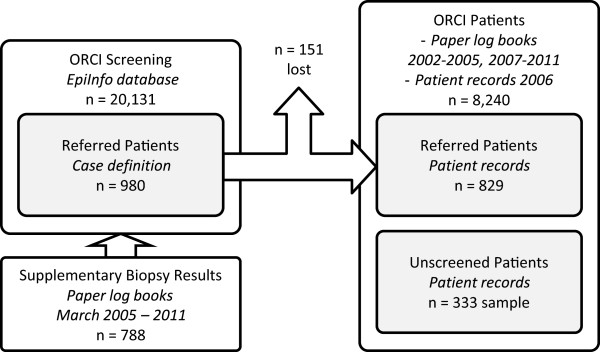
**Datasets.**

This retrospective cohort study was based on medical records from the ORCI cervical cancer screening clinic and the ORCI cancer treatment clinic (Figure 1), the only specialized cancer treatment center in Tanzania.

### Screening and referral populations

Each visit at the ORCI screening clinic is documented with a standardized paper reporting form and includes a screening clinic serial number, interview date, name, age, place of residence (municipality, district, or region), occupation, education, marital status, last menstrual period, age at marriage, gravidity, and screening clinic visit results. These forms were routinely entered into an EpiInfo database by ORCI staff, representing visits completed from the initiation of the screening program in November 2002 to June 2011 (n = 20,131). Only a couple of patients attended more than once during this period, so each form represents a unique person.

Since not all histopathology results had been entered into this database, especially in later years, we digitized the histopathology results from biopsy log books covering the periods March 2005 to October 2011 and merged them with the screening clinic database. The log book covering November 2002 to March 2005 was not available, but it appeared histopathology results from this time period had already been entered in the EpiInfo database. Of 1,001 results in these books, 788 were matched with the screening clinic database after eliminating duplicates, non-cervix biopsies, and results from outreach screening programs not conducted at ORCI.

The subset of women likely referred for treatment of invasive disease was identified and constitutes the referral population. As referrals from ORCI screening to treatment are not formally tracked, we developed a case definition to infer who was referred based on screening results and biopsy records. At ORCI, the screening clinic continues to operate under the protocols used in the original VIA screening study described above. Therefore, most women screened by VIA also undergo colposcopy, and most women with abnormal colposcopy findings are also biopsied for histopathology. A woman was considered referred (n = 980) if she was in the screening clinic database and had listed a final diagnosis of invasive cancer, a stage of invasive disease, a histopathology result of invasive cancer, or a colposcopy diagnosis of invasive cancer (unless contradicted by a histopathology result). While histopathology is the gold standard for diagnosis, not all patients with clinically obvious cancer receive a biopsy and not all biopsies are expeditiously processed, so we could not rely on histopathology alone to determine who was referred.

### Cervical cancer patient population

A dataset of all cervical cancer patients attending ORCI in this period was created by digitizing annual patient log books. The resulting dataset included medical records from November 2002 to October 2011, five months after the last observation in the ORCI screening clinic database, based on the expected limit of typical follow-up times. The 2006 patient log book was unavailable, so every patient record from 2006 (n = 2,549) was manually reviewed to identify cervical cancer cases. To improve the quality of the patient dataset, we identified 1,819 records where the log book was unclear on the cancer type. Of these records, 1,708 were located (93.9%) and reviewed to check for a cervical cancer diagnosis, which identified 200 additional cervical cancer cases (11.7%).

### Matching referrals to cervical cancer patients

To determine which women who attended the ORCI screening clinic and were referred for treatment of invasive cervical cancer followed up for at least one ORCI treatment clinic appointment, a SAS algorithm was developed to identify matches between referrals (n = 980) and the ORCI cervical cancer patient dataset (n = 8,240). All possible referral-patient combinations were generated, and then compared on name (asymmetric spelling distance), age, follow-up time, and place of residence. This process identified candidate matches for manual review (n = 7,198), while not excluding unlikely, but true matches. Manual review of potential matches identified a subset of 944 probable matches, of which 829 were determined to be true matches: 765 (92.3%) confirmed by reviewing patient records and 64 whose records were not reviewed but were extremely likely matches based on available information. Of the 765, 82.9% were confirmed by the presence of an ORCI screening clinic card in the record, which lists the screening clinic serial number, patient identifiers, screening clinic visit results, and follow-up instructions. 4.7% were missing the card but included the screening clinic serial number in the record, and 12.4% had neither. The follow-up status of those with neither was carefully reviewed to confirm a true match between screening and treatment datasets.

### Treatment assessment

Treatment data were abstracted for the 765 matches for whom a patient record was reviewed. Follow-up time was the difference between the screening clinic visit date and the first treatment appointment date. Treatment duration was the date difference between the first and last recorded clinical note. Attendance at another health facility before screening was assessed by clinical notes and presence of documents from other health facilities dated prior to the screening clinic visit.

The radiation dose prescribed (Gy), radiation dose completed (Gy), brachytherapy doses completed, and receipt of at least one dose of chemotherapy were abstracted from standardized treatment documentation forms. Radiation was considered complete if the woman received at least the dose she was prescribed, or if she completed at least 75% of her prescribed dose and her treatment duration was longer than 180 days (to account for prescriptions that were likely reduced). Women with no radiation prescription listed (n = 13) were considered to not have completed prescribed radiation because all had stage 1B disease or higher.

The screening clinic disease stage was from the screening clinic visit or, if missing, the clinical note of the first treatment appointment where follow-up time was no more than 15 days. The treatment clinic disease stage was from the clinical note of the first treatment appointment or, if missing, the screening clinic visit where follow-up time was no more than 15 days.

### Disease stage comparison

Records of a random sample (n = 333) of unscreened patients (n = 7,438) were successfully reviewed for disease stage, as assessed at first treatment appointment.

### Data analysis

The data were analyzed in SAS 9.3 (SAS Institute, Cary, NC). We compared characteristics of referred and non-referred women in the screening population using chi-squared and Mann-Whitney *U* tests. We then compared characteristics of women who followed up for at least one treatment clinic appointment to those lost to follow-up using chi-squared, Mann-Whitney *U*, and *t*-tests, followed by multivariable analysis with logistic regression. Among those who followed up, we compared characteristics of those who did and did not complete their prescribed radiation with the same methods. Finally, we assessed disease stage among women who attended the screening clinic and unscreened women by logistic regression.

The study was approved by the University of Michigan Health Sciences and Behavioral Sciences IRB and by the ORCI Academic, Research, Publications and Ethics Committee.

## Results

Among the 20,131 women who attended the screening clinic, 980 (4.9%) were diagnosed with invasive cancer (Table 1). This proportion increased over the study period, from less than 2% in 2002 and 2003 to more than 6% after 2005. Women diagnosed with invasive cancer were significantly more likely to be housewives or manual laborers, uneducated, widowed or separated, and to have had their last menstrual period more than 12 months ago. They were also significantly older, had younger age at marriage, and higher gravidity.

**Table 1:** **Demographic characteristics by invasive disease status among 20,131 women seen November 2002 to June 2011**

	No invasive cervical cancer		Invasive cervical cancer		
	N/mean	%/SD	N/mean	%/SD	
**Total**	19,151	95.1	980	4.9	
**Occupation**					
Housewife	9,847	51.4%	755	77.0%	p < 0.0001^*^
Manual	727	3.8%	64	6.5%	
Technical	235	1.2%	2	0.2%	
Office going	2,126	11.1%	32	3.3%	
Teaching	1,332	7.0%	11	1.1%	
Professional	1,217	6.4%	3	0.3%	
Business	3,529	18.4%	100	10.2%	
Other	101	0.5%	9	0.9%	
*Missing*	37	0.2%	4	0.4%	
**Education**					
None	1,723	9.0%	411	41.9%	p < 0.0001^*^
Primary	10,365	54.1%	481	49.1%	
Middle	4,524	23.6%	63	6.4%	
High School	628	3.3%	9	0.9%	
College	1,843	9.6%	9	0.9%	
*Missing*	68	0.4%	7	0.7%	
**Marital status**					
Married/regular partner	15,551	81.2%	630	64.3%	p < 0.0001^*^
Widowed	1,368	7.1%	207	21.1%	
Separated	1,402	7.3%	131	13.4%	
Other	677	3.5%	6	0.6%	
*Missing*	153	0.8%	6	0.6%	
**Last menstrual period**					
<12 months ago	15,962	83.3%	530	54.1%	p < 0.0001^*^
>12 months ago	3,060	16.0%	436	44.5%	
*Missing*	129	0.7%	14	1.4%	
**Age**	39.4	10.0	50.5	12.9	p < 0.0001^**^
*Missing*	48		3		
**Age at marriage**	21.4	4.4	19.0	4.3	p < 0.0001^**^
*Missing*	1,966		94		
**Gravidity**	3.8	2.7	6.3	3.2	p < 0.0001^**^
*Missing*	731		15		
**Year of screening**					
2002	549	2.9%	10	1.0%	p < 0.0001^*^
2003	4,681	24.4%	77	7.9%	
2004	2,465	12.9%	82	8.4%	
2005	1,570	8.2%	81	8.3%	
2006	1,089	5.7%	92	9.4%	
2007	1,610	8.4%	134	13.7%	
2008	1,698	8.9%	144	14.7%	
2009	2,258	11.8%	143	14.6%	
2010	2,363	12.3%	155	15.8%	
2011	866	4.5%	58	5.9%	
*Missing*	2	0.0%	4	0.4%	

*Chi-squared test.

**Mann-Whitney *U* test (*t* approximation).

**Figure 2:**
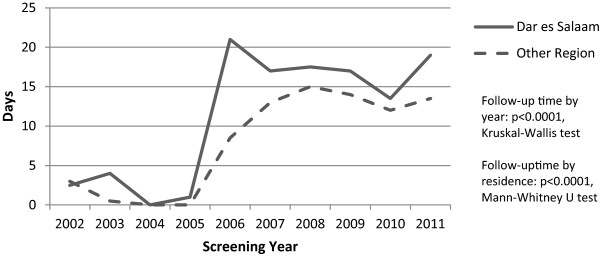
**Median follow-up time by year.**

**Figure 3:**
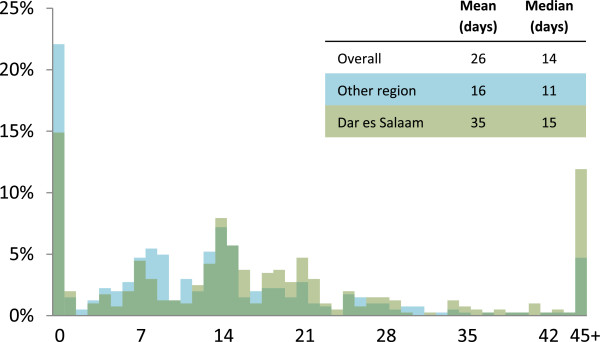
**Histogram of follow-up time.**

Among the 980 with invasive cancer, 829 (84.6%) followed up at ORCI for at least one treatment clinic appointment (Table 2). This proportion did not change significantly over the study period. The mean and median follow-up times were 26 and 14 days, respectively. The median follow-up time increased significantly over the study period (Figure 2), and was longer for women from Dar es Salaam (Figures 2 and 3). Women with a skilled occupation, more than primary education, last menstrual period less than 12 months ago, younger age, lower gravidity, residence in Dar es Salaam, and earlier disease stage at screening were significantly less likely to follow up. Marital status and age at marriage were not significantly associated with follow-up status.

**Table 2:** **Demographic and disease characteristics by follow-up status among 980 women referred for treatment**

	Lost to follow-up		Followed up at ORCI		
	N/mean	%/SD	N/mean	%/SD	
**Total**	151	15.4	829	84.6	
**Occupation**					
Housewife	103	68.2%	652	78.6%	p = 0.0008^*^
Manual	8	5.3%	56	6.8%	
Skilled/Other	40	26.5%	117	14.1%	
*Missing*	0	0.0%	4	0.5%	
**Education**					
None	48	31.8%	363	43.8%	p = 0.0042^*^
Primary	83	55.0%	398	48.0%	
>Primary	20	13.2%	61	7.4%	
*Missing*	0	0.0%	7	0.8%	
**Marital status**					
Married/regular partner	108	71.5%	522	63.0%	p = 0.0557^*^
Widow/separated/other	43	28.5%	301	36.3%	
*Missing*	0	0.0%	6	0.7%	
**Last menstrual period**					
<12 months ago	96	63.6%	434	52.4%	p = 0.0107^*^
>12 months ago	53	35.1%	383	46.2%	
*Missing*	2	1.3%	12	1.4%	
**Age**	46.9	13.4	51.1	12.7	p = 0.0002^***^
**Age at marriage**	19.1	3.7	18.9	4.4	p = 0.2271^**^
**Gravidity**	5.6	3.2	6.4	3.2	p = 0.0022^***^
**Year of screening**					
2002-2003	13	8.6%	74	8.9%	p = 0.4405^†^
2004-2005	23	15.2%	140	16.9%	
2006-2007	35	23.2%	191	23.0%	
2008-2009	42	27.8%	245	29.6%	
2010-2011	38	25.2%	175	21.1%	
*Missing*	0	0.0%	4	0.5%	
**Place of residence**					
Dar es Salaam	96	63.6%	417	50.3%	p = 0.0027^*^
Other Region	55	36.4%	412	49.7%	
**Screening clinic disease stage**					
1	32	21.2%	69	8.3%	p < 0.0001^†^
2	36	23.8%	313	37.8%	
3	13	8.6%	248	29.9%	
4	0	0.0%	10	1.2%	
*Missing*	70	46.4%	189	22.8%	

*Chi-squared test.

**Mann-Whitney *U* test (*t* approximation).

***t-test with equal variances.

^†^Mantel-Haenszel chi-squared test for trend.

In multivariable models, education, timing of last menstrual period, gravidity, and place of residence were no longer significantly associated with follow-up status after adjustment for age and disease stage (Table 3). In a final model including age, disease stage, occupation, and place of residence, women with a skilled occupation were half as likely (OR = 0.51, 95% CI 0.28-0.91) to have followed up compared to housewives. Each one-unit increase in disease stage was associated with a 2.70 fold (95% CI 1.87-3.89) increase in the odds of follow-up. Each ten-year increase in age was associated with a 19 percent increase (OR = 1.19, 95% CI 0.96-1.48) in the odds of follow-up, although the CI included one. Residence outside of Dar es Salaam was associated with a 1.47 fold (95% CI 0.87-2.48) increase in the odds of follow-up compared to women from Dar es Salaam, but again, the CI included one.

**Table 3:** **Logistic regression of follow-up status among 980 women referred for treatment**

	Crude		Age-adjusted		Age, stage-adj.		Final Model ^*^	
	OR	CI	OR	CI	OR	CI	OR	CI
**Occupation**								
Housewife	1		1		1		1	
Manual	1.11	0.51-2.39	1.16	0.53-2.50	1.04	0.35-3.14	0.96	0.32-2.92
Skilled/Other	0.46	0.31-0.70	0.58	0.38-0.90	0.47	0.27-0.84	0.51	0.28-0.91
**Education**								
None	1		1		1			
Primary	0.63	0.43-0.93	0.82	0.54-1.24	0.99	0.56-1.75		
>Primary	0.40	0.22-0.73	0.53	0.29-0.99	0.68	0.30-1.54		
**Marital status**								
Married/regular partner	1		1		1			
Widow/separated/other	1.45	0.99-2.12	1.21	0.81-1.80	1.09	0.63-1.88		
**Last menstrual period**								
<12 months ago	1		1		1			
>12 months ago	1.60	1.11-2.30	1.01	0.63-1.62	1.12	0.60-2.09		
**Age**								
Continuous (10 year)	1.31	1.13-1.52			1.31	1.07-1.61	1.19	0.96-1.48
**Age at marriage**								
Continuous	0.99	0.95-1.03	1.00	0.96-1.04	1.00	0.94-1.06		
**Gravidity**								
Continuous	1.09	1.03-1.16	1.06	0.99-1.13	1.07	0.97-1.17		
**Place of residence**								
Dar es Salaam	1		1		1		1	
Other Region	1.73	1.21-2.47	1.57	1.09-2.27	1.62	0.97-2.70	1.47	0.87-2.48
**Screening clinic disease stage**								
Continuous	3.16	2.21-4.52	2.91	2.02-4.18			2.70	1.87-3.89

*Final model adjusted for age, screening clinic disease stage, place of residence, and occupation.

Of the 829 women who followed up for at least one treatment clinic appointment, 765 were assessed for treatment received. Of these, 687 (89.8%) received at least some radiation, brachytherapy, or chemotherapy (Table 4) and 633 (82.8%) completed their prescribed dose of radiation (Table 5). Of all women diagnosed with invasive disease at screening, women prescribed less than 40Gy radiation and those with later disease stage at treatment were significantly less likely to complete radiation. Women who were older, treated in a later study year, and resided in Dar es Salaam were less likely to completion radiation, but these bivariable associations were not significant.

**Table 4:** **ORCI treatment received among 765** ^*^ **women with invasive cervical cancer who followed up for treatment**

	No chemotherapy				≥1 dose chemotherapy			
	Brachytherapy doses				Brachytherapy doses			
Prescribed radiation	0	1	2-3	Total	0	1	2-3	Total
Received none	78	0	0	78	1	0	0	1
Partially completed	50	0	0	50	3	0	0	3
Completed	445	3	49	497	101	4	31	136
**Total**	573	3	49	625	105	4	31	140

*64/829 missing ORCI treatment data.

**Table 5:** **Patient characteristics by radiation completion among 829 women who followed up for treatment at ORCI**

	Did not complete prescribed radiation		Completed prescribed radiation		
	N/mean	%/SD	N/mean	%/SD	
**Total**	132	17.3	633	82.8	
	*Missing n = 64*				
**Occupation**					
Housewife	106	80.3%	499	78.8%	p = 0.2996^*^
Manual	12	9.1%	41	6.5%	
Skilled/Other	14	10.6%	93	14.7%	
**Education**					
None	64	48.5%	278	43.9%	p = 0.5405^*^
Primary	57	43.2%	308	48.7%	
>Primary	10	7.6%	45	7.1%	
*Missing*	1	0.8%	2	0.3%	
**Marital status**					
Married/regular partner	81	61.4%	400	63.2%	p = 0.6607^*^
Widow/separated/other	51	38.6%	231	36.5%	
*Missing*	0	0.0%	2	0.3%	
**Last menstrual period**					
<12 months ago	66	50.0%	332	52.4%	p = 0.5802^*^
>12 months ago	65	49.2%	294	46.4%	
*Missing*	1	0.8%	7	1.1%	
**Age**	53.2	14.2	51.0	12.5	p = 0.1027^†^
**Age at marriage**	19.2	5.8	18.8	3.8	p = 0.9204^**^
**Gravidity**	6.2	3.4	6.5	3.1	p = 0.3472^***^
**Year of first treatment appointment**					
2002-2003	6	4.5%	61	9.6%	p = 0.0571^‡^
2004-2005	24	18.2%	107	16.9%	
2006-2007	23	17.4%	145	22.9%	
2008-2009	43	32.6%	177	28.0%	
2010-2011	36	27.3%	143	22.6%	
**Place of residence**					
Dar es Salaam	74	56.1%	302	47.7%	p = 0.0808^*^
Other Region	58	43.9%	331	52.3%	
**Treatment clinic disease stage**					
1	9	6.8%	67	10.6%	p = 0.0221^‡^
2	58	43.9%	333	52.6%	
3	57	43.2%	212	33.5%	
4	5	3.8%	21	3.3%	
*Missing*	3	2.3%	0	0.0%	
**Attended another health facility before screening**					
Yes	25	18.9%	150	23.7%	p = 0.2528^*^
No	106	80.3%	483	76.3%	
*Missing*	1	0.8%	0	0.0%	
**Prescribed ≥40 Gy radiation**					
Yes	105	79.5%	569	89.9%	p = 0.0008^*^
No	27	20.5%	64	10.1%	
**Follow-up time (days)**	23.4	42.1	23.3	77.4	p = 0.3284^**^

*Chi-squared test.

**Mann-Whitney *U* test (*t* approximation).

***t-test with equal variances.

^†^ *t*-test with unequal variances.

^‡^Mantel-Haenszel chi-squared test for trend.

In a multivariable model including age, disease stage, place of residence, and treatment year, each one-unit increase in disease stage was associated with a 28 percent decrease (OR = 0.72, 95% CI 0.55-0.96) in the odds of radiation completion (Table 6). Each two-year increase in treatment year was associated with a 15 percent decrease (OR = 0.85, 0.72-1.00, p = 0.0499) in the odds of radiation completion. Residence outside of Dar es Salaam was associated with a 1.46 fold (95% CI 0.99-2.15) increase in the odds of radiation completion compared to women from Dar es Salaam, however the CI included one. A ten-year increase in age was associated with a 13 percent decrease (OR = 0.87, 95% CI 0.75-1.01) in the odds of radiation completion, but the CI included one.

**Table 6:** **Logistic regression of radiation completion among 829 women who followed up for treatment at ORCI**

	Crude		Age-adjusted		Age, stage-adj.		Final Model ^**^	
	OR	CI	OR	CI	OR	CI	OR	CI
**Occupation**								
Housewife	1		1		1			
Manual	0.73	0.37-1.43	0.72	0.36-1.41	0.68	0.34-1.34		
Skilled/Other	1.41	0.78-2.57	1.28	0.69-2.36	1.15	0.62-2.14		
**Education**								
None	1		1		1			
Primary	1.24	0.84-1.84	1.10	0.72-1.68	1.09	0.71-1.67		
>Primary	1.04	0.50-2.17	0.88	0.41-1.89	0.75	0.35-1.65		
**Marital status**								
Married/regular partner	1		1		1			
Widow/separated/other	0.92	0.62-1.35	1.01	0.67-1.51	0.98	0.66-1.48		
**Last menstrual period**								
<12 months ago	1		1		1			
>12 months ago	0.90	0.62-1.31	1.20	0.75-1.93	1.25	0.77-2.01		
**Age**								
Continuous (10 year)	0.88	0.76-1.01			0.89	0.77-1.03	0.87	0.75-1.01
**Age at marriage**								
Continuous	0.98	0.94-1.02	0.97	0.93-1.01	0.97	0.93-1.02		
**Gravidity**								
Continuous	1.03	0.97-1.09	1.07	1.00-1.14	1.06	0.99-1.13		
**Year of first treatment appointment**								
Continuous (2 year)	0.85	0.73-1.00^*^	0.85	0.72-1.00^*^	0.85	0.73-1.00	0.85	0.72-1.00^*^
**Place of residence**								
Dar es Salaam	1		1		1		1	
Other Region	1.40	0.96-2.04	1.48	1.01-2.16	1.44	0.98-2.13	1.46	0.99-2.15
**Treatment clinic disease stage**								
Continuous	0.73	0.55-0.96	0.74	0.56-0.97			0.72	0.55-0.96
**Attended another health facility before screening**								
No	1		1		1			
Yes	1.32	0.82-2.11	1.31	0.82-2.10	1.31	0.81-2.11		
**Prescribed ≥40 Gy radiation** ^***^								
No	1		1		1			
Yes	2.29	1.39-3.75	2.17	1.32-3.59	1.88	1.08-3.25		
**Follow-up time**								
Continuous (1 week)	1.00	0.98-1.02	1.00	0.98-1.02	1.00	0.98-1.02		

*Significant at the α = 0.05 level, CI includes 1.00 due to rounding.

**Final model adjusted for age, year of first treatment appointment, place of residence, and treatment clinic disease stage.

***Prescribed **≥**40 Gy radiation was not included in the final model because it is an intermediate variable between disease stage and radiation completion.

The median treatment duration was 380 days (mean 600, standard deviation 615) among those who completed their prescribed radiation, and 0 days (mean 40, standard deviation 116) among those who did not complete their prescribed radiation (0 indicating completion of only the initial appointment at the treatment clinic). Follow-up time did not significantly differ between those who did and did not complete their prescribed radiation. All women assessed were symptomatic at their first appointment. A quarter (23%) of women who followed up had attended another health facility before attending the ORCI screening clinic regarding their symptoms.

Among the sample of unscreened women who presented for treatment at ORCI, 7.8% (26/333) had stage 1 disease. Among women referred for treatment from the screening clinic who had stage information available, 14.0% (101/721) had stage 1 disease. Attending the screening clinic was associated with 1.61 (95% CI 1.01-2.56) times the odds of stage 1 disease, after adjusting for age and place of residence (Table 7).

**Table 7:** **Factors associated with stage 1 disease among screened** ^*^ **and unscreened women**

	Crude		Final model ^**^	
	OR	CI	OR	CI
**Screening status**				
Unscreened	1		1	
Screened	1.92	1.22-3.02	1.61	1.01-2.56
**Age**				
Continuous (10 year)	0.79	0.68-0.93	0.83	0.71-0.97
**Place of residence**				
Dar es Salaam	1		1	
Other Region	0.50	0.34-0.73	0.59	0.40-0.87

*Disease stage assessed at screening, before any loss to follow-up.

**n = 719 screened and n = 333 unscreened in final model.

## Discussion

This study is the first study to evaluate treatment completion following referral from a VIA screening program for invasive cervical cancer. Our results show a high proportion of women referred from the screening clinic followed up and completed treatment for their cancer at ORCI. We estimate at least 76% of women diagnosed with invasive disease at the ORCI screening clinic received at least some treatment, and at least 70% completed their prescribed radiation. These proportions are likely higher, were we to account for treatment received elsewhere.

Lower disease stage, having a skilled occupation, residence in Dar es Salaam, and younger age were independently associated with loss to follow-up. Higher disease stage, residence in Dar es Salaam, older age, and later year of first treatment appointment were independently associated with incomplete treatment among those who followed up. Significantly more screened women had stage 1 disease (14.0%) than unscreened women (7.8%). In most cases, the screening clinic appears to facilitate diagnosis and treatment, rather than screening, for women with invasive cervical cancer.

We found that more advanced disease stage was associated with greater likelihood of follow-up. However, women with more advanced disease stage were less likely to complete their prescribed radiation. The south Indian trial suggested invasive cancer (all stages combined) was associated with a greater likelihood of treatment completion compared to low grade, pre-cancerous lesions (OR = 1.55, 95% CI 0.7-3.6) [15], but this is not directly comparable to our study because we did not include women with pre-cancerous lesions. Furthermore, the south Indian trial did not separately assess follow-up and treatment completion among those who followed up, as in our study.

Some of those lost to follow-up likely received treatment elsewhere. Women with skilled occupations were half as likely to follow up for treatment as housewives or manual laborers. This likely reflects that women with higher socioeconomic status have the means to travel outside of Tanzania for cancer treatment, typically to Kenya, South Africa, or India. Occupation was not, however, associated with completion of radiation among those who did follow up at ORCI. This finding suggests finances were not a significant barrier to treatment, which is government funded and provided free of charge in Tanzania. The south Indian trial also found occupation was not associated with compliance to treatment, which was free of charge [15].

ORCI is the only specialized cancer treatment center in Tanzania, so it attracts patients from every region of the country. Nearly half of women with invasive disease were from outside of Dar es Salaam, and they were more likely to both follow up and complete treatment compared to women residing in Dar es Salaam. Residents of other regions also presented for follow-up sooner than women from Dar es Salaam and were less likely to have stage 1 disease. The reasons for these differences by region should be explored in future studies.

Older women were more likely to follow up, but were less likely to complete radiation therapy, although these associations were not significant in multivariable analysis. The effect on follow-up appears to be driven by loss to follow-up among the youngest patients, while the effect on radiation appears to be driven by incomplete treatment among the oldest patients (results not shown).

Although the proportion of women who followed up did not change significantly over the study period, the data suggest that the proportion of women completing radiation decreased over time. The volume of patients (all cancers) increased 64% over the study period, from 1,897 in 2002 to 3,102 in 2011, placing an increased burden on radiation therapy equipment, which sometimes broke down. Scheduling radiation therapy also became increasingly difficult, since many patients required 15 to 20 sessions to complete their prescribed dose. The increased patient volume may also account for the increase in follow-up time seen over the study period, since follow-up time is a function of both provider (supply) and patient (demand) factors.

The assessment of treatment focused on radiation because it was most consistently documented and is the primary cervical cancer treatment modality at ORCI. However, for patients treated with curative intent, radiation is supplemented by brachytherapy when the machine is operable, and chemotherapy in some cases. Only women who completed their prescribed radiation went on to receive brachytherapy, per ORCI guidelines. Less than one-fifth of women received at least one dose of chemotherapy, and this was often for palliation.

The proportion of screened women diagnosed with invasive cancer (4.9%) was higher than reported in other African studies (3.5% in Mali [11], 1.9% in Zambia [9], and 0.6% in Angola [13]). This difference likely reflects both the high burden of cervical cancer in Tanzania, as well as the program design. While the absolute number of cervical cancer cases referred from the screening clinic each year did double from 2003-2010, the increasing proportion of women diagnosed with invasive disease was also a function of significant changes in the number attending the screening clinic over time. Initially, considerable efforts were taken to recruit women from the community for screening [12], with 4,758 women screened during the first full year of the program (2003). The lowest proportion of invasive disease was seen in that year, since many of the women screened would not have attended unless invited. In contrast, women with invasive cervical cancer or another gynecologic problem are typically symptomatic and more likely to attend the screening clinic uninvited. These women represented a larger proportion of screened women in later years, when there were fewer screening promotion efforts in the community.

It is clear that women with symptomatic cervical cancer are attending the ORCI screening clinic seeking a proper diagnosis, rather than screening. They have often been treated for symptoms of cervical cancer at the regional and local level, delaying a formal diagnosis and initiation of appropriate therapy in many cases. In this study, all women who followed up were symptomatic, and a quarter of women who followed up had already been seen elsewhere. ORCI’s VIA screening program has directly contributed to development of the human capital and clinic infrastructure necessary for not only VIA screening, but also for providing basic gynecologic care and referral. With these resources, women can be efficiently directed to appropriate care.

Screening also resulted in a higher likelihood of diagnosis with stage 1 disease, compared to unscreened women treated at the same institution. This downstaging suggests the ORCI screening program is not only facilitating diagnosis for women with more advanced, symptomatic disease, but also detection for women with earlier stage, less symptomatic or asymptomatic disease. This may be the case for some of the 32 women with stage 1 disease who were lost to follow-up.

A limitation of the study was the lack of a common identification number used throughout all records, potentially leading to missed cases in our analysis. Although a screening clinic serial number partially filled this purpose, it was not always used in patient records and was not included in patient log books. Referrals from screening to treatment were not formally tracked, so a case definition had to be established based on available data that may have misclassified some screened women. Since we only reviewed treatment records at ORCI, we could not account for the small proportion of women who may have been treated at institutions in other countries. Finally, these results may not be generalizable to screening programs operated distant from cervical cancer treatment services.

An important strength of this study is the inclusion of 980 cases of cervical cancer referred from a VIA screening program over an 8.5 year period, nearly twice the number of women included in the next largest study [11]. This sample size permitted evaluation of follow-up and treatment completion across the diverse demographics served by the screening clinic, with a wide range of occupations, education levels, and ages represented. Additionally, most patient and disease characteristics were measured before follow-up and treatment. This is the first study to differentiate between follow-up and treatment completion and we have demonstrated that the factors associated with these outcomes appear to differ.

Future studies could investigate what proportion of women diagnosed with invasive cervical cancer at ORCI screening receives treatment elsewhere. In addition, research using patient interviews could help explain the patterns of follow-up and treatment completion seen in this study.

## Conclusions

Our study shows that in addition to detecting and treating pre-cancerous lesions, a VIA screening program can effectively facilitate diagnosis and timely treatment of invasive cancer. Furthermore, our results offer new insights into why women with invasive cervical cancer might be lost to follow-up or fail to complete treatment after being referred from a VIA screening program in a low-resource setting. Although they represent a small proportion of women screened in these programs, women with invasive disease stand to greatly benefit from proper detection, diagnosis, and treatment. Consideration of these findings may improve efforts to intervene before loss to follow-up and improve outcomes for these women.
